# Reading tea leaves or tracking true constructs? An assessment of personality-based latent profiles in eating disorders

**DOI:** 10.3389/fpsyt.2024.1376565

**Published:** 2024-05-14

**Authors:** Helo Liis Soodla, Kärol Soidla, Kirsti Akkermann

**Affiliations:** ^1^ Institute of Psychology, University of Tartu, Tartu, Estonia; ^2^ Centre for Cognitive and Behavioural Therapy, Tartu, Estonia

**Keywords:** personality profiles, eating disorder subtypes, latent profile analysis, cluster analysis, perfectionism, impulsivity

## Abstract

**Background:**

Eating disorder (ED) subtyping studies have often extracted an undercontrolled, an overcontrolled and a resilient profile based on trait impulsivity and perfectionism. However, the extent to which methodological choices impact the coherence and distinctness of resulting subtypes remains unclear.

**Objective:**

In this paper, we aimed to assess the robustness of these findings by extracting personality-based subtypes on a sample of ED patients (*N* = 221) under different analytic conditions.

**Methods:**

We ran four latent profile analyses (LPA), varying the extent to which we constrained variances and covariances during model parametrization. We then performed a comparative analysis also including state ED symptom measures as indicators. Finally, we used cross-method validation via k-means clustering to further assess the robustness of our profiles.

**Results:**

Our results demonstrated a four-profile model based on variances in impulsivity and perfectionism to fit the data well. Across model solutions, the profiles with the most and least state and trait disturbances were replicated most stably, while more nuanced variations in trait variables resulted in less consistent profiles. Inclusion of ED symptoms as indicator variables increased subtype differentiation and similarity across profiles. Validation cluster analyses aligned most with more restrictive LPA models.

**Conclusion:**

These results suggest that ED subtypes track true constructs, since subtypes emerged method-independently. We found analytic methods to constrain the theoretical and practical conclusions that can be drawn. This underscores the importance of objective-driven analytic design and highlights its relevance in applying research findings in clinical practice.

## 1 Introduction

Personality traits play a significant role in eating disorders (EDs), interacting with state symptomatology and impacting treatment prognosis (1, 2). This interplay has been investigated by creating personality-based typologies. Subtyping emphasizes the importance of dimensional traits in meaningfully interpreting clinical phenotypes: they can make explanations more substantial and help tailor treatment. Among various personality traits, impulsivity and perfectionism have arguably received the most attention among ED samples (3). We take impulsivity to reflect a trait-level disposition toward rapid reactions to varied stimuli, which can lead to both unwanted (inadequate deliberation, insufficient perseverance) and wanted (quick and adaptive responding) outcomes (4–6). We interpret perfectionism as a tendency to base one’s self-worth on achievements which are, in turn, defined by high personal standards and the desire to avoid mistakes (7, 8).

Based on these traits, researchers have identified three main personality subtypes that reflect varied levels of self-regulation among people with EDs: the overcontrolled, the undercontrolled and the resilient profile (3). The overcontrolled profile features high perfectionism, rigidity and inhibition; the undercontrolled profile impulsivity, disinhibition and emotional reactivity, while the resilient profile exhibits limited personality disturbances [e.g (9)].

However, previous reviews of personality subtypes in EDs have shown heterogeneity due to various factors (3). These include differences in sample composition (e.g. only anorexia nervosa (AN), bulimia nervosa (BN) or binge eating disorder (BED), or transdiagnostic samples), sample sizes, research settings and statistical methods (3). This variability makes generalizing findings to different populations challenging. Two additional reasons for non-convergence emerge. Firstly, the threshold for extracting and identifying discrete personality profiles differs across studies. This leads to fuzzy subtype borders and difficulties in comparing results (10). Secondly, since impulsivity and perfectionism are multidimensional traits, the meanings of “undercontrol” and “overcontrol” become context-dependent (10). This can result in the 3-profile model being overfitted to demonstrate alignment with previous results (10).

Examination of methodological choices can showcase reasons behind the discrepancies. Subtyping studies often utilize person-centered methods, most commonly latent profile analysis [LPA (11, 12)]. LPA is a probabilistic mixture modelling method used to construct latent patterns based on observed variance in continuous indicator variables (13–15). LPA is a theory-informed model-based method that assumes that any covariation among indicator variables should be accounted for by the extracted latent variables (conditional independence; 16). Researchers determine the number of profiles to be extracted and model fit is estimated using different indices (17). Person-centered research has also used cluster analysis (CA): a set of classification procedures aiming to construct subgroups based on observed variances in clustering variables (18). In comparison to LPA, CA is not reliant on a pre-specified measurement model and aims to optimize clusters to align with the inputted data structure (18).

Personality-based LPAs on ED samples have yielded varied results, detecting 3–6 profiles (9, 19–25). Differences have arisen upon extracting smaller additional classes with either combined impulsivity and perfectionism [e.g (23)] or facets of undercontrol/overcontrol (24, 25).

This divergence raises the question of whether the well-documented triadic model tracks the existence of three true dimensional constructs or stems partly from methodological artefacts. Three points of potential methodological bias are highlighted.

Firstly, LPA parametrization choices impact pattern detection (26–28). LPA axiomatically presumes conditional independence and in many software programs, variable variances are fixed to be equal across profiles and covariances are set to 0 (15, 27, 29). While this approach results in parsimonious models and shorter computation time, it might not reflect psychiatric constructs that are often correlated, leading to misleading descriptions (26, 30). Simulation studies have demonstrated model misspecification to result in overextraction of classes (27). Additionally, the suitability and accuracy of fit indices depends on mathematical model specifications (27). Given that these methodological decisions are often undisclosed, their impact on results remains unclear (30–32).

Secondly, discordant findings can stem from the use of different indicator or clustering variables for subtype construction. Previous studies have employed various personality inventories, some tapping specific traits, some aiming to describe personality or temperament more broadly (19–23). Recently, the level of indicator data has also been shown to be significant – composite scores might fail to encapsulate underlying measurement models, further contributing to biased estimates (33). While an increased number of indicators can enhance model convergence, inclusion of individual items might result in uninterpretable solutions in smaller samples (34, 35).

Thirdly, there is ongoing debate about whether ED symptom measures should be included as indicator variables [e.g (25)] or as external validators of the derived subtypes (9, 19–21, 23). This choice hinges on both the theoretical model underlying the postulated state-trait interactions, and data analytic choices. If personality and ED symptoms are viewed as mutually influencing each other, then separation into distinct state- and trait-models might be unmerited – two-way causation complicates the distinction of causal antecedents and consequences (36). However, there is also support in favor of competing models that do not presuppose a bidirectional interaction (37). Similarly, there are methodological advantages and disadvantages to including ED symptoms as indicators. Inclusion of more theoretically relevant indicators can lead to stronger and more interpretable associations between the latent variable and distal outcomes (34, 38). However, incorporation of symptoms derived from categorical classification systems can hinder progress towards a bottom-up dimensional theoretical space.

A review of previous literature reveals no clear consensus on recommended methodology. This raises the question: to what extent do these analytical nuances really matter? This study aims to address these gaps by examining the similarities and differences between personality-based subtypes that have been extracted under different methodological conditions. We derived 8 models based on 2 sets of indicators (symptoms excluded and included) and 4 model parametrization options. As such, our objective is to refine and validate an existing theory, as opposed to proposing more competing solutions or an entirely new measurement model, e.g. a network-based conceptualization (39). Such a latent variable approach holds potential to, upon repeated and longitudinal validation, move from descriptions to explanations. To assess our solutions’ robustness and provide cross-method replicability, we used CA post-extraction (40). Previous studies have primarily used constrained LPAs and not included ED symptoms as indicator variables. To ease interpretation, we took this model to serve as a baseline that we compared other models against (12). However, we do not suggest this model to be conceptually superior.

Based on previous research, we hypothesized that the derived models would reflect the self-regulation model with undercontrolled, overcontrolled and resilient personality subtypes. Additionally, we expected inclusion of ED symptoms as indicator variables to further differentiate subtypes. Literature on the impact of methodology is scarce, however, we did also anticipate that models would be comparable and profiles overlapping across analytic conditions. Furthermore, we predicted that the more constrained and parsimonious LPA models would align more with CA validation results, and that less-constrained models would exhibit more non-convergence [for previous non-ED overlap studies (41, 42)].

## 2 Method

### 2.1 Participants

The sample comprised 249 women with a primary diagnosis of an ED, with a mean age of 21.91 (*SD* = 6.78). In total, 48.2% of the participants had been diagnosed with AN, 43.8% with BN and 6.8% with BED; participants had *M* = 2.54 comorbid diagnoses (*Mdn* = 2). On average, they reported their ED as having lasted for 4.4 years (*Mdn* = 3 years; minimum 2 months, maximum 30 years); illness duration was highly correlated with age (*r* = .72). Exclusion criteria included intellectual disability, acute psychotic episodes, and involuntary hospitalization.

Participants were recruited and completed assessment in an inpatient setting at the Psychiatric Clinic of the Tartu University Hospital. Data were collected by trained clinical psychologists. Participants’ written informed consent was obtained after the nature of the procedures had been fully explained. Study design was reviewed and approved by the Research Ethics Committee of the University of Tartu (243/T-20, 196/T-17). Data used in this research is available upon request and can be accessed in line with participants’ informed consent.

### 2.2 Measures

#### 2.2.1 Psychiatric diagnoses

Psychiatric diagnoses were established using the Mini-International Neuropsychiatric Interview [M.I.N.I 5.0.0 (43)]. Clinical interviews were conducted by trained licensed clinical psychologists and diagnoses were confirmed by treating psychiatrists.

#### 2.2.2 Perfectionism

Perfectionism was assessed with Frost’s Multidimensional Perfectionism Scale [FMPS (44)]. FMPS is a self-report questionnaire with items rated on a 5-point scale, the Estonian version has 28 items (45). The adapted questionnaire’s factor structure resembles the original FMPS, items make up four subscales: concern over mistakes and doubts about actions (concern over mistakes), excessive concern with parents’ expectations and evaluation (parental standards), excessively high personal standards (personal standards) and concern with precision, order and organization [organization (45)]. The first two scales reflect negative perfectionism, the latter two positive facets of perfectionism. The internal consistency of the subscales varied between α = .82–.95. (ω = .83–.95; fit in a principal factor analysis (PFA)). Subscale scores were used as indicator variables.

#### 2.2.3 Impulsivity

Trait impulsivity was assessed with Dickman’s Impulsivity Inventory [DII (6)]. DII is a self-report questionnaire with items rated on a 5-point scale, the Estonian version has 24 items, making up two subscales: functional and dysfunctional impulsivity (46). The internal consistency of both subscales was α = .85 (PFA ω = .85–.86). Subscale scores were used as indicator variables.

#### 2.2.4 Eating disorder symptoms

ED symptoms were assessed with the Eating Disorder Assessment Scale [EDAS (47)]. EDAS is a self-report questionnaire with 29 items rated on a 6-point scale, making up four subscales: restrained eating, binge eating, purging, and preoccupation with weight and body image. The internal consistency of the subscales varied between α = .91–.96. (PFA ω = .92–.96). Subscale scores were used as indicator variables.

### 2.3 Data analysis

Data were first assessed for missingness. All individuals (*n* = 28) whose data were missing across all subscales of at least one indicator measure were excluded from the sample. Given the limited data available for these participants and the relatively small sample size, imputation might not accurately represent the population of interest (48, 49). Two individuals’ data were missing at random on one indicator subscale. Due to violating the multivariate normality assumption, MissForest non-parametric imputation was used to impute these data (50). Analyses were run with 1000 random trees and the out-of-bag imputation error was found satisfactory at NRMSE = 0.29. Imputed values were rounded up to the first whole number.

To evaluate our hypotheses, 1–8-profile LPA models were assessed under four conditions based on the constriction of variances and covariances:

(1) Model 1: equal variances and covariances fixed to 0;
(2) Model 2: varying variances and covariances fixed to 0;
(3) Model 3: equal variances and equal covariances;
(4) Model 4: varying variances and varying covariances (26–28).

Allowing for varying variances and covariances curtails the conditional independence criterion and enables assessment of parametrization’s impact on model choice (29).

All analyses were first run on personality indicators; a second set of analyses was performed with ED symptom measures included.

To avoid converging on local maxima, we increased random starting value sets to 1000 and 250; the maximum number of initial stage iterations was fixed at 20. Model fit was assessed using several fit indices. We primarily relied on the Bayesian Information Criterion (BIC) and the sample-adjusted Bayesian Information Criterion (SABIC), as previous research has demonstrated their superior performance (51–54). While Akaike’s Information Criterion (AIC) can have lower pattern detection accuracy, it remains useful in small samples where meaningful subgroups include few people (53, 54). Entropy estimates, which quantify the accuracy of classification, were used as rule-of-thumb heuristics (53). Values surpassing .80 indicate good fit (53). Additionally, the fit of k versus k–1 profile models was assessed using the bootstrapped likelihood ratio test (BLRT, 20 bootstrap draws) which has shown to be a more reliable test in comparison to other bootstrap methods (53). Finally, models were assessed for parsimony and theoretical interpretability (17, 35).

After conducting LPAs, the best fitting models were validated via k-means clustering. K-means clustering is a type of iterative partitioning clustering that attempts to derive cohesive and non-overlapping clusters by assigning datapoints to the closest cluster centroid based on Euclidean distance, thus minimizing within-cluster variance (55, 56). The number of clusters was predetermined by the LPA results. Data were scaled and subjected to 25 different random starting assignments to stabilize the cluster solution (56).

Data analyses were performed in R 4.1.1. using the mclust, MplusAutomation (Mplus version 6.12 was used via R), NbClust, ggplot2 and circlize packages (15, 57–61).

## 3 Results

The final sample comprised 221 individuals (*M*_age_= 21.46, *SD*_age_= 6.53; *M*_illness_duration_ = 4.35, *SD*_illness_duration_ = 4.73; 49.3% AN, 43.0% BN and 7.7% BED). Descriptive statistics are presented in **Supplementary Table 1**.

### 3.1 Profile extraction

The results of the personality-only and the symptoms-included sets of LPAs are presented in **Tables 1** and **2**, respectively. Upon running the LPAs, R produced warning messages without indicating errors, highlighting the necessity of further analysis of results. To avoid confirmation bias, we will refer to the extracted profiles with their numbers, instead of labelling the subtypes (10).

**Table 1:** Fit indices for 1–8 profile LPAs for personality-only models under four parametrization conditions.

No		LL	Entropy	BIC	AIC	SABIC	BLRT	BLRT (*p*)	Smallest profile *%*
Equal variances and covariances fixed to 0									
1		–4,499.38	—	9,063.53	9,022.75	9,025.50	—	—	—
2		–4,424.21	.76	8,950.99	8,886.42	8,890.78	150.33	<.001	44.3%
3		–4,396.12	.77	8,932.58	8,844.23	8,850.19	56.19	<.001	23.5%
**4**		**–4,366.02**	**.80**	**8,910.18**	**8,798.04**	**8,805.60**	**60.19**	**<.001**	**14.9%**
5		–4,351.81	.78	8,919.54	8,783.61	8,792.78	28.42	<.001	13.1%
6		–4,338.28	.81	8,930.27	8,770.55	8,781.32	27.06	<.001	3.6%
7		–4,322.02	.82	8,935.54	8,752.03	8,764.41	32.52	.01	5.4%
8		–4,309.60	.84	8,948.49	8,741.20	8,755.17	24.84	.07	0.9%
Varying variances and covariances fixed to 0									
1		–4,499.38	—	9,063.53	9,022.75	9,025.50	—	—	—
2		–4,393.57	.91	8,922.10	8,837.15	8,842.87	211.61	<.001	21.7%
3		–4,333.93	.87	8,872.99	8,743.86	8,752.57	119.28	<.001	6.3%
**4**		**–4,299.87**	**.90**	**8,875.04**	**8,701.74**	**8,713.42**	**68.13**	**<.001**	**6.3%**
5		–4,272.98	.83	8,891.43	8,673.95	8,688.62	54.45	<.001	6.3%
6		–4,249.49	.86	8,914.63	8,652.97	8,670.62	53.13	.06	5.8%
7		–4,229.17	.86	8,944.17	8,638.33	8,658.95	46.42	.07	6.3%
8		–4,220.00	.87	8,996.02	8,646.01	8,669.61	36.87	.38	2.3%
Equal variances and equal covariances									
1		–4,384.38	—	8,914.51	8,822.76	8,828.95	—	—	—
2		–4,354.68	.86	8,892.90	8,777.37	8,785.16	59.40	<.001	30.3%
3		–4,333.16	.86	8,887.65	8,748.32	8,757.71	43.05	<.001	10.9%
**4**		**–4,314.93**	**.85**	**8,888.97**	**8,725.85**	**8,736.85**	**36.47**	**<.001**	**4.5%**
5		–4,302.40	.83	8,901.71	8,714.81	8,727.41	25.05	.06	4.1%
6		–4,291.66	.84	8,919.01	8,707.32	8,721.53	21.48	.18	4.1%
7		–4,278.60	.87	8,929.68	8,695.21	8,711.02	26.12	<.001	2.3%
8		–4,266.30	.87	8,942.87	8,684.61	8,702.02	24.60	.09	2.3%
Varying variances and varying covariances									
1		–4,384.38	—	8,914.51	8,822.76	8,828.95	—	—	—
2		–4,298.57	.99	8,894.05	8,707.15	8,719.75	171.62	<.001	9.5%
3		–4,244.22	.88	8,936.49	8,654.44	8,673.46	108.71	<.001	6.3%
**4**		**–4,203.70**	**.91**	**9,006.59**	**8,629.39**	**8,654.82**	**78.47**	**.17**	**4.1%**
5		–4,171.87	.90	9,094.09	8,621.74	8,653.59	52.67	.60	6.3%
6		–4,145.00	.92	9,191.50	8,624.00	8,662.27	32.16	1.00	4.1%
7		*NA*	*NA*	*NA*	*NA*	*NA*	*NA*	*NA*	*NA*
8		–4,097.50	.91	9,398.78	8,640.99	8,692.09	117.03	.50	3.2%

4-profile model in bold. One computation resulted in an error (NA). No, number of classes in model; LL, log likelihood; BIC, Bayesian Information Criterion, AIC, Akaike’s Information Criterion; SABIC, sample-adjusted Bayesian Information Criterion; LMR, adjusted Lo–Mendell–Rubin likelihood ratio test; BLRT, bootstrap likelihood ratio test.

**Table 2:** Fit indices for 1–8 profile LPAs for symptoms-included models under four parametrization conditions.

No		LL	Entropy	BIC	AIC	SABIC	BLRT	BLRT (*p*)	Smallest profile *%*
Equal variances and covariances fixed to 0									
1		–7,771.16	—	15,650.28	15,582.32	15,586.90	—	—	—
2		–7,631.38	.85	15,430.09	15,324.75	15,331.85	279.567	<.001	29.9%
3		–7,566.41	.85	15,359.55	15,216.83	15,226.45	129.923	<.001	25.9%
**4**		**–7,534.64**	**.84**	**15,355.38**	**15,175.28**	**15,187.43**	**63.544**	**<.001**	**19.9%**
5		–7,504.46	.90	15,354.39	15,136.91	15,151.58	60.371	<.001	3.6%
6		–7,477.84	.88	15,360.54	15,105.68	15,122.87	53.229	<.001	3.1%
7		–7,454.87	.89	15,373.98	15,081.73	15,101.44	45.949	<.001	6.3%
8		–7,429.63	.89	15,382.88	15,053.25	15,075.48	50.480	<.001	4.1%
Varying variances and covariances fixed to 0									
1		–7,771.16	—	15,650.28	15,582.32	15,586.90	—	—	—
2		–7,557.86	.94	15,337.04	15,197.72	15,207.11	426.60	<.001	26.2%
3		–7,463.09	.94	15,260.86	15,050.17	15,064.38	189.54	<.001	6.3%
**4**		**–7,394.64**	**.94**	**15,237.33**	**14,955.28**	**14,974.30**	**136.89**	**<.001**	**6.3%**
5		–7,331.11	.91	15,223.62	14,870.22	14,894.04	122.69	<.001	5.8%
6		–7,287.72	.94	15,250.21	14,825.44	14,854.08	91.27	<.001	5.8%
7		–7,261.60	.94	15,311.34	14,815.21	14,848.66	35.33	1.00	7.7%
8		–7,237.45	.92	15,376.39	14,808.90	14,847.16	27.01	1.00	6.3%
Equal variances and equal covariances									
1		–7,489.40	—	15,329.68	15,108.80	15,123.69	—	—	—
2		–7,443.16	90	15,296.57	15,038.31	15,055.72	92.49	<.001	34.8%
3		–7,404.00	.91	15,277.64	14,982.00	15,001.93	78.31	<.001	16.3%
**4**		**–7,372.44**	**.92**	**15,273.90**	**14,940.88**	**14,963.33**	**63.12**	**<.001**	**15.8%**
5		–7,350.35	.94	15,289.09	14,918.69	14,943.67	44.19	<.001	13.6%
6		–7,336.88	.91	15,321.53	14,913.75	14,941.25	26.94	1.00	3.6%
7		–7,316.33	.93	15,339.82	14,894.66	14,924.68	34.90	.11	3.6%
8		–7,298.41	.92	15,363.36	14,880.82	14,913.35	34.33	.02	3.6%
Varying variances and varying covariances									
1		–7,489.40	—	15,329.68	15,108.80	15,123.69	—	—	—
2		–7,331.56	.95	15,370.28	14,925.12	14,955.13	315.69	<.001	24.9%
3		–7,224.79	.95	15,513.02	14,843.58	14,888.71	213.54	<.001	5.8%
**4**		**–7,143.40**	**.95**	**15,706.51**	**14,812.80**	**14,873.06**	**201.65**	**<.001**	**19.5%**
5		–7,102.45	.97	15,980.90	14,862.90	14,938.28	86.85	.67	9.1%
6		–7,037.11	.98	16,206.49	14,864.22	14,954.72	101.51	1.00	5.0%
7		–7,010.39	.97	16,509.32	14,942.77	15,048.39	182.74	1.00	5.0%
8		–6,944.56	.97	16,733.95	14,943.12	15,063.86	180.22	<.001	<.1%

4-profile model in bold. No, number of classes in model; LL, log likelihood; BIC, Bayesian Information Criterion, AIC, Akaike’s Information Criterion; SABIC, sample-adjusted Bayesian Information Criterion; LMR, adjusted Lo–Mendell–Rubin likelihood ratio test; BLRT, bootstrap likelihood ratio test.

In the personality-only analyses, the absolute values of log likelihood (LL) decrease consistently across parametrization conditions, predicting the 8-profile model to be best-fitting. AIC and SABIC showed similar trends across Models 1, 2 and 3. In Model 4, AIC and SABIC values indicate the 5-profile model to describe the data the best. There is more variance in BIC estimates. Under the constrained Model 1, BIC predicts the 4-profile solution to be best-fitting; under Models 2 and 3, it flags 3–4-profile solutions. Value differences between these solutions remain trivial. BIC points to a 2-profile solution being best-fitting under Model 4. Since the second-best solution only has one profile, this can suggest underprediction.

Entropy values approximate or surpass the .80 benchmark in nearly all solutions, rendering this indicator relatively unhelpful in determining model fit. Similarly, the BLRT estimates yield unsatisfactory power to distinguish between solutions. Regardless, significant BLRT *p*-values indicate that solutions with a smaller number of profiles, 1–4, are generally preferred.

Across models, BIC, AIC and SABIC values were generally comparable, with Models 2 and 3 performing slightly better based on the BIC, and Models 2 and 4 following AIC and SABIC. Non-convergence emerged for the 7-profile solution (Model 4) due to the model’s instability.

Similar trends emerged upon running the analyses with ED measures included. Models 1–3 were comparable, as the BIC indicated 3–5-profile solutions to fit the data the best and the absolute values of LL and AIC decreased with added number of profiles extracted. Model 4 also highlighted the 4-profile model as best-fitting. Similarly to the personality-only analyses, Models 2 and 4 appeared to fit the data slightly better based on the AIC and SABIC, while the BIC indicated Models 2 and 3 to be the best-fitting.

The 4-profile model was chosen for further investigation across all 8 analytic conditions. While evidence for best-fitting model was mixed, several indicators that did distinguish between profile solutions sufficiently flagged the 4-profile solution. Since no compelling evidence arose for the 1–2 and 7–8 profile solutions, we decided against using them as the basis for comparisons of invariance, as parsimony might reduce clinical usefulness and high complexity lead to less interpretability.

### 3.2 Comparison of profiles

**Figure 1:**
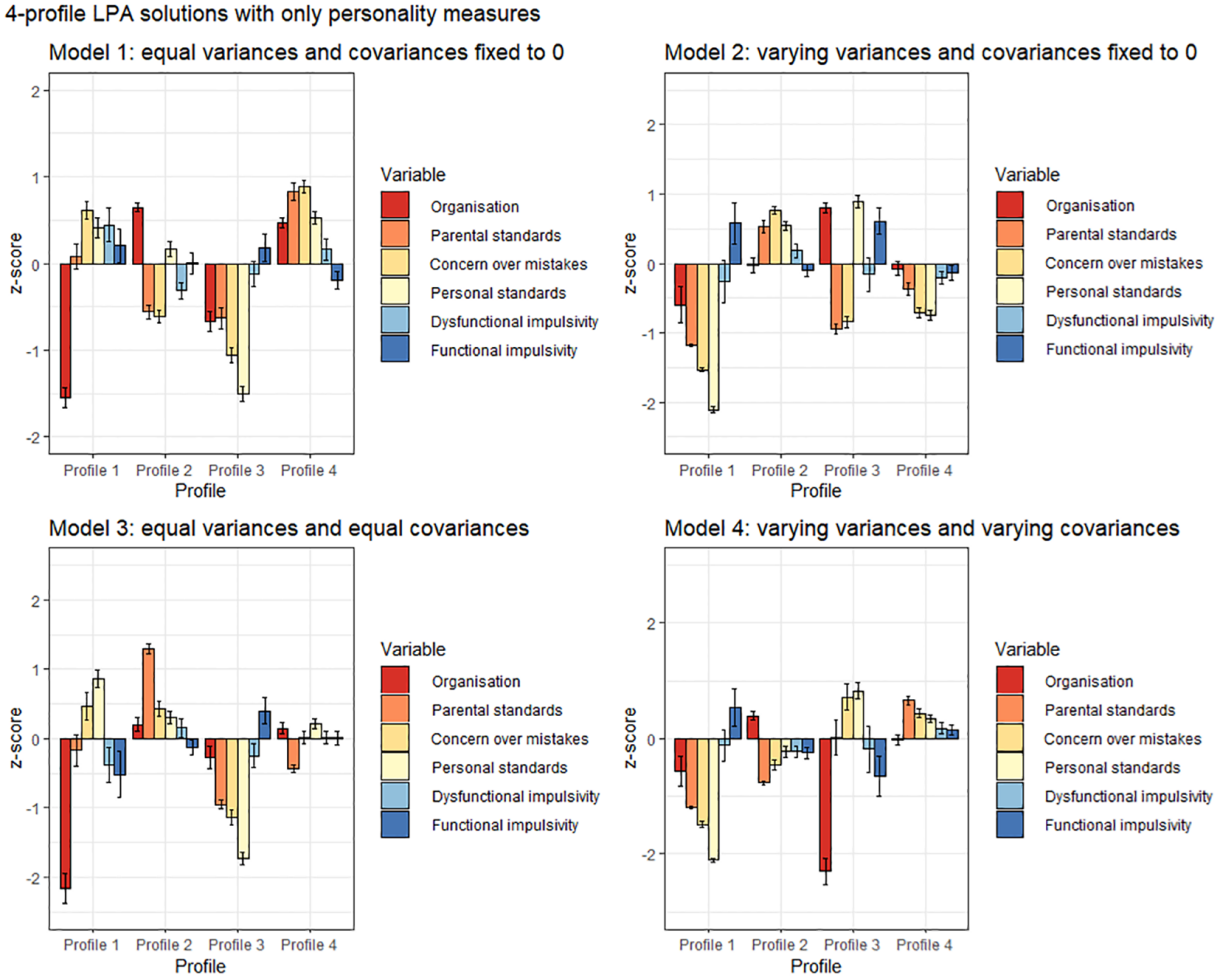
Personality-based 4-profile LPA solutions across four analytical conditions. Confidence intervals depict standard errors.

The 4-profile personality-only models are presented in **Figure 1**. Regardless of analysis parametrization, a profile with low perfectionism, low dysfunctional impulsivity and heightened functional impulsivity arose (Model 1, Profile 3; Model 2, Profile 1; Model 3, Profile 3; Model 4, Profile 1). There was more variance across the other profiles. In Models 1, 2 and 4, profiles differentiated by high positive perfectionism were extracted (respectively, Profile 2, Profile 3 and Profile 2), in Model 3, levels of high positive perfectionism covaried with increased negative perfectionism, or remained near-average (Profile 1 and Profile 4). Model 1 produced a profile characterized by elevated dysfunctional impulsivity and low organization (Profile 1), purely impulsive classes were less pronounced under other analytic conditions.

**Figure 2:**
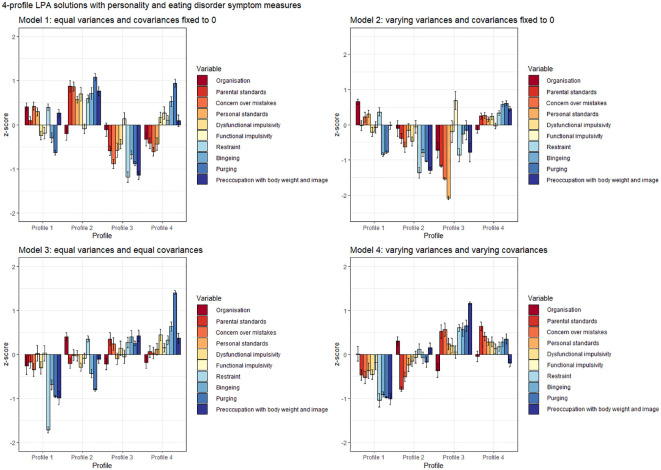
Personality and eating disorder symptoms 4-profile LPA solutions across four analytical conditions. Confidence intervals depict standard errors.

Addition of ED symptoms resulted in distinct trends across models (see **Figure 2**). Firstly, the profile with low scores on all indicator measures besides functional impulsivity (Model 1, Profile 3; Model 2, Profile 3; Model 3, Profile 1) remained intact. Models 2 (Profiles 2 and 3) and 4 (Profiles 1 and 2) detected two profiles with less pronounced overall psychopathology, as opposed to one. Secondly, a profile with high perfectionism, high impulsivity and elevated scores on ED measures emerged in all models but was most pronounced in Models 1 (Profile 2) and 2 (Profile 4) – in Model 3, perfectionism scores were less elevated (Profile 4) and in Model 4, low organization was more pronounced than high impulsivity (Profile 3). Thirdly, in Models 1–3 (respectively, Profile 1, Profile 1 and Profile 2), a profile with high perfectionism and high restrictive ED pathology was detected. Finally, Model 1 (Profile 4) and Model 3 (Profile 4) pointed towards the emergence of a highly impulsive and purging profile. A trend towards a similar profile (Profile 3) was apparent in Model 4, however, other ED measures besides purging were also elevated.

### 3.3 Predictive power of personality-only profiles

We assessed the extent to which personality-only profiles predicted ED scores to further evaluate if adding ED symptoms as indicator variables was merited. Due to violations of normality, Kruskal-Wallis tests were run. For Model 1, the effect sizes for comparisons of means across different profiles were *η2* = .03–.10 for different ED pathology measures; for Model 2 *η2* = .04–.10, Model 3 *η2* = .01–.10 and Model 4 *η2* = .04–.10. Overall, profile categorization most sufficiently predicted variation in restraint scores (moderate effect of *η2* = .10) for all models.

### 3.4 Validation of profiles via K-means clustering

**Figure 3:**
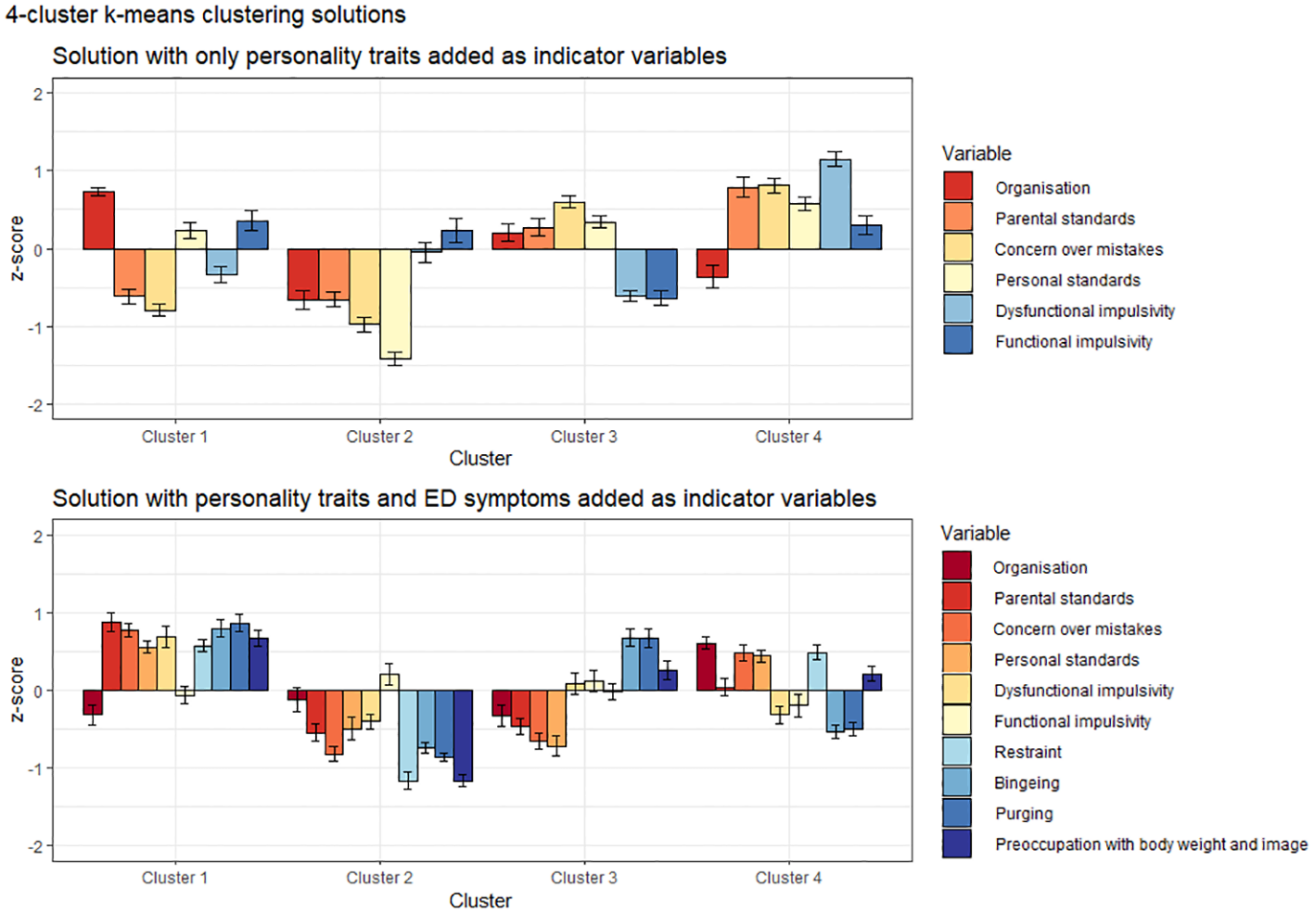
Cluster analytic solutions for personality-only and symptoms-added models. Confidence intervals depict standard errors.

LPA findings were validated via k-means clustering. Results are presented in **Figure 3**.

Personality-only clusters generally mirrored subtypes emerging via LPA. A cluster with low trait pathology was detectable (Cluster 2). Cluster 4 was characterized by combined high impulsivity and high perfectionism, while the two remaining clusters were mainly distinguished by varying levels of positive (Cluster 1) and negative perfectionism (Cluster 3).

When ED symptoms were added, we observed the emergence of a low pathology group (Cluster 2), as well as a subtype of individuals with low trait pathology but elevated ED symptoms (Cluster 3). One cluster was characterized by overall high trait and ED pathology (Cluster 1) and one by predominantly overcontrolled-restrictive pathology (Cluster 4). As such, the cluster solutions were similar to the most constrained LPAs under Model 1 parametrization conditions.

### 3.5 Overlap between extracted profiles and clusters

Since constrained models are more often reported on (26), and our LPA Model 1 was easily interpretable and had larger profile sizes that allowed for more informative comparisons, it was chosen as basis for calculations of subtype overlap.

**Figure 4:**
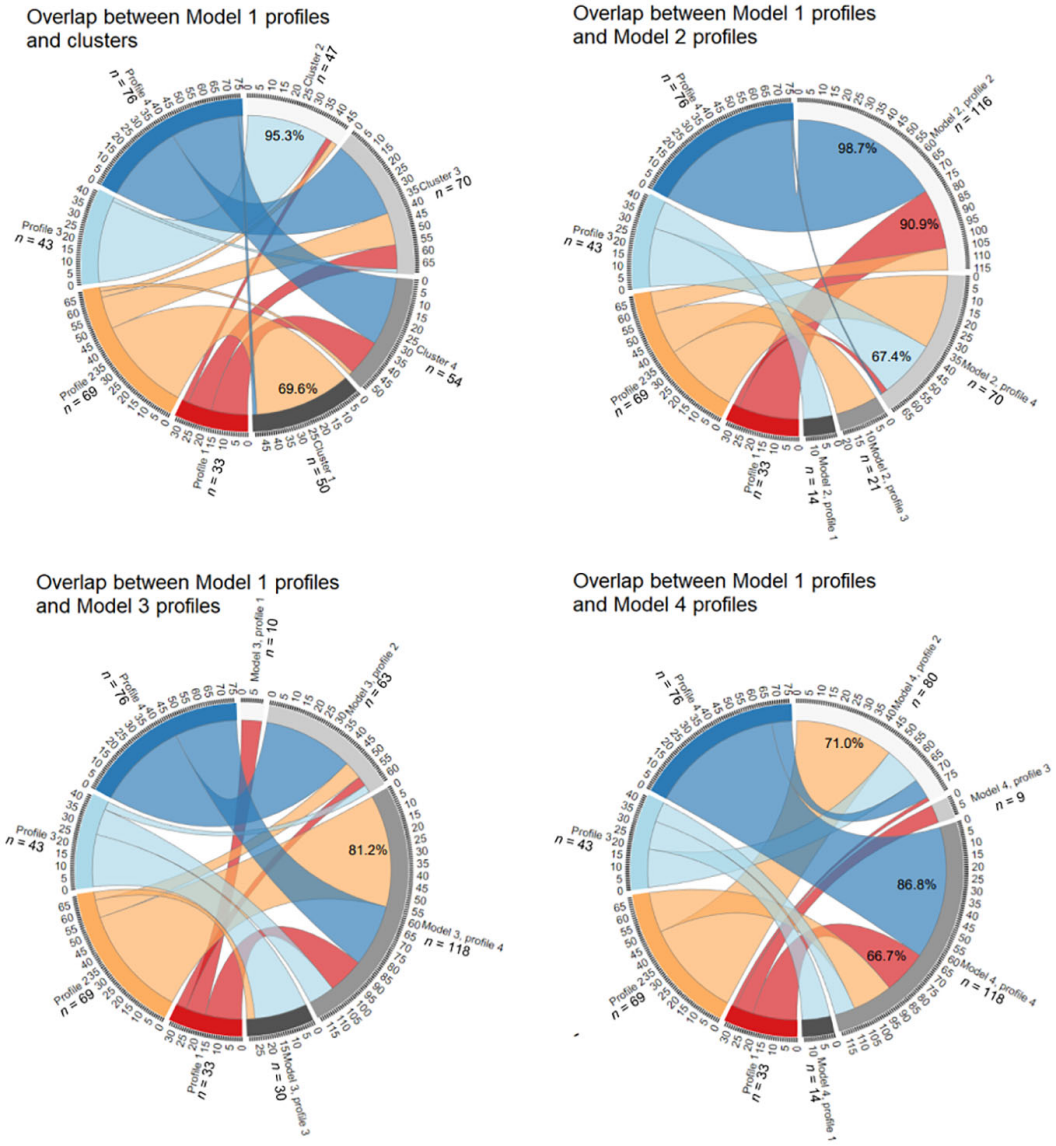
Overlap between personality-only LPA Model 1 and the k-means model, LPA Model 2, LPA Model 3 and LPA Model 4. See **Supplementary Material** for an interactive .html file.

Classification overlap and the size of all subtypes for personality-only models is depicted in **Figure 4**. Profile 1 with low organization and high impulsivity overlapped significantly with Profile 2 of Model 2 (90.9%), however, across other parametrizations where subtypes were more equally sized, this profile divided into 2–3 different profiles. The positively perfectionistic Profile 2 overlapped with Cluster 1 (69.6%), Profile 4 of Model 3 (81.2%) and Profile 2 of Model 4 (71.0%). Profile 3 – individuals with low pathology – overlapped with Cluster 2 (95.3%) and Profile 4 of Model 2 (67.4%), however, participants in this profile were divided into separate subtypes under parametrization conditions of Models 3–4. The largest Profile 4 with heightened overall perfectionism and above-average impulsivity aligned with Profile 2 of Model 2 (98.7%, however, this profile was disproportionately large) and Profile 4 of Model 4 (86.8%).

**Figure 5:**
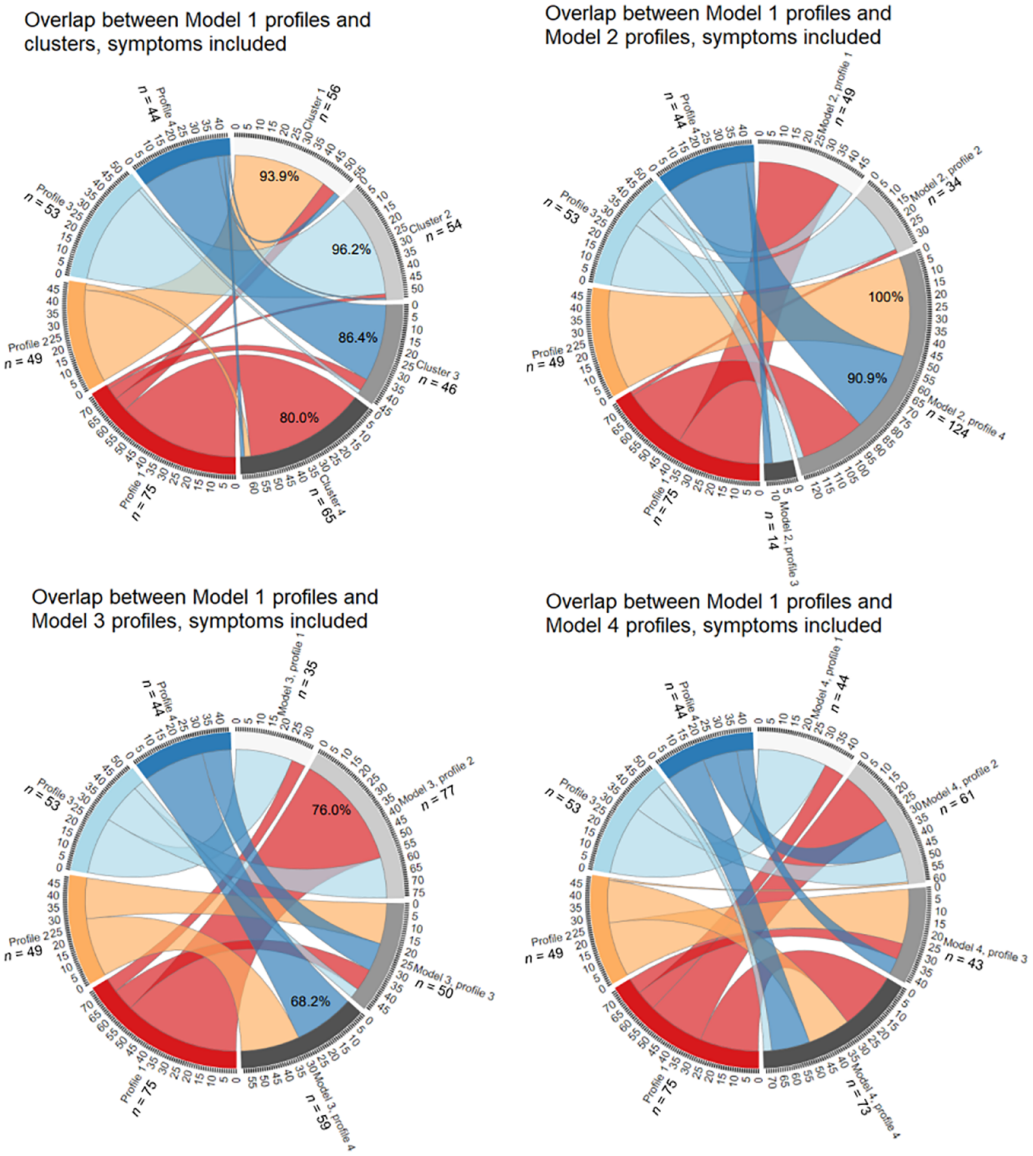
Overlap between symptoms-included LPA Model 1 and the k-means model, LPA Model 2, LPA Model 3 and LPA Model 4. See **Supplementary Material** for an interactive .html file.

**Figure 5** depicts overlap between symptoms-added models. Profile 1 with high perfectionism, restraint and preoccupation overlapped significantly with Cluster 4 (80.0%) and Profile 2 of Model 3 (76.0%). Profile 2 with elevated trait and eating pathology was also replicated, with 93.9% overlap with Cluster 1. While all participants of Profile 2 of Model 1 fell into Profile 4 of Model 2, this resulted from the latter profile being disproportionately large (also encompassing 90.9% of Profile 4). Low pathology Profile 3 of Model 1 was relatively robust, demonstrating 96.2% overlap with Cluster 2 and aligning with Profile 2 of Model 2 (60.4%) and Profile 1 of Model 4 (64.2%). The impulsive and bingeing-purging Profile 4 aligned well with Cluster 3 (86.4%), and profile 4 of Model 3 (68.2%).

## 4 Discussion

This study aimed to assess how methodological choices influence personality subtypes among individuals with EDs. Our findings support a self-regulation-based model and identified a 4-subtype solution as best-fitting, with impulsivity and perfectionism playing key roles in distinguishing between the profiles. We found inclusion of ED symptoms as indicators to help differentiate subtypes and increase subtype overlap. Our second hypothesis regarding model comparability found partial support, with some profiles overlapping significantly and others being relatively unique to model configurations. Finally, cluster analytic validation analyses aligned the most with constrained model parametrization, while less-constrained models were also less stable.

While we refrained from labelling our profiles to prevent bias, the detection of a 4-subtype personality-based model generally aligns with prior studies that have postulated a high impulsivity, a high perfectionism, and a resilient class (19, 22, 23). Under several configurations, we observed the emergence of a fourth, combined perfectionism-impulsivity subtype which has previously been demonstrated in both non-clinical populations and on ED samples (62, 63).

Comparing the different personality-only LPA models, Model 1 with equal variances and covariances coerced to be zero yielded the most equally sized profiles. Other parametrization conditions led to disproportionately small or large profiles. Model 1 also aligned the best with cluster analysis results. Varying subtype sizes across models contributed to reduced overlap between them. Both positively and negatively perfectionistic profiles arose, while impulsivity impacted profile differentiation less, possibly because we used only two subscales to reflect this construct. In the personality-only model, the resilient individuals with limited trait pathology were detected most reliably. Previous studies on non-ED populations have shown that 2- and 3-profile models that quantitatively distinguish between low and high disturbance can fit data well [e.g., for exercise behavior (64); trait impulsivity (65); cognition (66)]. It is possible that even if qualitatively distinct profiles are not robustly detectable, personality-based profiling can indicate which groups of individuals display high and low trait psychopathology.

Inclusion of ED symptoms increased differentiation among profiles. While personality profiles characterized by high organization and personal standards were concealed by the variation of ED symptoms’ contribution to profile differentiation, the impact of impulsive traits was more pronounced under the symptoms-included model. Generally, subtypes with low personality pathology also demonstrated low state symptomatology. Profiles with higher levels of perfectionism tended to be further characterized by restraint and preoccupation. Profiles with more pronounced impulsivity had high scores on all ED symptom measures, especially purging. Importantly, if ED symptomatology was included, extracted subtypes became more robust and overlap between models increased: profiles with low overall pathology, heightened overall pathology and heightened internalizing pathology were reliably replicated. Furthermore, including ED symptom measures proved useful, as personality-only subtypes had limited power in predicting ED symptomatology. These results mirror those in (62) who found personality profiles to explain 5–8% of variance in ED symptoms.

This study has several important limitations. Firstly, the relatively small sample size for LPA may have limited our ability to detect true classes and precluded item-based profile construction (53). Sample size constraints also made subgroup analyses impossible. Secondly, we did not compare model solutions across external validation measures, opting for a more data analytic validation design in alignment with our study objectives. Additionally, while we used k-means clustering to validate LPA models, future studies could explore fuzzy clustering approaches that help detect probabilities of belonging to a specific cluster and thus ensure better correspondence to the LPA methodology (67). Finally, due to an ethnically homogenous sample of women, the cross-cultural generalizability of our findings requires further research. Despite these limitations, we believe this study to introduce a novel methodology-focused perspective on ED subtyping studies.

In summary, our results underscore the robustness of impulsivity- and perfectionism-based subtypes, while highlighting that more nuanced profile characteristics are dependent on methodological choices. This variability is reflected in fit indices operating differently across different models, subtype sizes varying and the smaller resultant profiles diverging qualitatively. So, there is proof of the bottom-up constructed profiles tracking real entities, but methodology’s impact is significant. Failure to report specific results instead of general labels can conceal this influence. For instance, if our personality-only Model 1 Profile 1 was deemed the “undercontrolled” class in virtue of individuals’ low organization and elevated dysfunctional impulsivity, heightened levels of some facets of perfectionism would gain no attention. Or, if an individual was grouped in Profile 1 under symptoms-included Model 1 and labelled “overcontrolled and restricting”, it would be missed that under Model 2 parametrization, 42.7% of these individuals fall under a profile that demonstrated heightened binging-purging symptoms as well. While subtype labelling eases dissemination of results and can, at heuristic level, contribute to meaningful interpretations, we believe the risk of overfitting models to be high.

Based on these findings, we present two key recommendations for subtyping research. Firstly, profiling methodology should be derived from study objectives, data characteristics and the criteria for choosing best-fitting models. These choices should be transparently reported. We showed more constrained LPA models to produce more equally-sized and more easily interpretable profiles, while the relaxation of analytic constraints resulted in less cohesive solutions but allowed to account for potential conditional dependence. Furthermore, personality-only subtyping enabled the delineation of multidimensional trait constructs, while the addition of symptoms as indicators provided added explanatory power in terms of conceptualizing the entire clinical phenotype. Either approach could be suitable, if in alignment with study objectives. Secondly, given the divergent yet complementary results, different model solutions should be presented comparatively. For example, if a constrained model is chosen as reflecting the data structure the best, using this as baseline for later analyses can introduce additional bias. However, if covariances are allowed and variances are not coerced to be equal, non-convergence can hinder choosing models with more relaxed parametrization as baseline. In the future, latent variable models could be supplemented with network and other machine learning approaches to further elucidate the associations between variables of interest, without subscribing to causal interpretations (39, 68). Regardless, we believe a demonstration of the robustness of latent variable models to contribute to theory-building and offer input for network variable selection – to arrive at better descriptions of psychopathology in line with the principle of epistemic iteration (69–71).

We also believe that these findings hold clinical significance. Identifying if people align with an overcontrolled, undercontrolled, resilient, or combined impulsivity-perfectionism profile can help determine most suitable treatment options and tailor assessments to target the personality-level mechanisms that maintain disordered eating (72, 73). However, our research underscores that caution should be taken when generalizing from subtype to individual clinical presentations, and points towards the utility of idiographic approaches to integrating personality and psychopathology (74). In clinical practice, this suggests the importance of routinely assessing personality traits for both treatment matching and outcome assessment. To further validate the existence of subtype entities that are independent of methodology and have predictive and explanatory value, self-report measures of personality traits could be supplemented by behavioral or physiological markers reflective of trait-level disturbance [e.g., impulsivity (5)]. Additionally, incorporating more clinical variables, such as history of traumatic life events and longitudinal data about illness development and severity trajectories would increase results’ generalizability across the ED spectrum.

## Data availability statement

The data supporting the conclusions of this article will be made available by the authors upon request and in line with participants’ informed consent.

## Ethics statement

The studies involving humans were approved by Research Ethics Committee of the University of Tartu. The studies were conducted in accordance with the local legislation and institutional requirements. The participants provided their written informed consent to participate in this study.

## Author contributions

HLS: Conceptualization, Data curation, Formal analysis, Investigation, Methodology, Visualization, Writing – original draft, Writing – review & editing. KS: Conceptualization, Data curation, Formal analysis, Investigation, Methodology, Visualization, Writing – original draft, Writing – review & editing. KA: Conceptualization, Data curation, Formal analysis, Investigation, Methodology, Project administration, Resources, Supervision, Writing – original draft, Writing – review & editing.
