# Supplementary material for: Reading tea leaves or tracking true constructs? An assessment of personality-based latent profiles in eating disorders
Source: Front Psychiatry. 2024 May 14;15:1376565. doi: 10.3389/fpsyt.2024.1376565 (PMC11130490; doi:10.3389/fpsyt.2024.1376565)
Supplement: Supplementary file 1 [file DataSheet_1.zip › Supplementary Table.docx]

Supplementary Material

Reading tea leaves or tracking true constructs? An assessment of personality-based latent profiles in eating disorders

Helo Liis Soodla^*^, Kärol Soidla, Kirsti Akkermann

*** Correspondence:** Helo Liis Soodla: helo.liis.soodla@ut.ee

**Supplementary Table 1**

*Descriptive statistics for the total initial dataset and the dataset with no missing values*

| Variable | Total sample | | | Sample with no missing values (*n* = 221) | |
| --- | --- | --- | --- | --- | --- |
|  | *n* | *M* | *SD* | *M* | *SD* |
| Organization | 224 | 21.37 | 4.94 | 21.43 | 4.88 |
| Concern over mistakes | 224 | 12.45 | 7.82 | 12.43 | 7.78 |
| Personal standards | 224 | 17.75 | 7.71 | 17.71 | 7.73 |
| Parental standards | 224 | 9.27 | 7.54 | 9.28 | 7.55 |
| Functional impulsivity | 242 | 20.66 | 8.19 | 20.63 | 8.17 |
| Dysfunctional impulsivity | 242 | 16.64 | 7.77 | 16.82 | 7.82 |
| Restraint | 244 | 22.78 | 10.12 | 23.00 | 10.39 |
| Bingeing | 245 | 18.83 | 11.58 | 18.63 | 11.73 |
| Purging | 244 | 7.30 | 6.72 | 7.17 | 6.81 |
| Preoccupation with weight and body image | 245 | 24.11 | 11.06 | 24.16 | 11.21 |

*Note.* No statistically significant differences emerged between initial total dataset and dataset with no missing values.
