# Supplementary material for: Reading tea leaves or tracking true constructs? An assessment of personality-based latent profiles in eating disorders
Source: Front Psychiatry. 2024 May 14;15:1376565. doi: 10.3389/fpsyt.2024.1376565 (PMC11130490; doi:10.3389/fpsyt.2024.1376565)
Supplement: Supplementary file 1 [file DataSheet_1.zip › Supplementary Figures.docx]

Supplementary Material

Reading tea leaves or tracking true constructs? An assessment of personality-based latent profiles in eating disorders

Helo Liis Soodla^*^, Kärol Soidla, Kirsti Akkermann

*** Correspondence:** Helo Liis Soodla: helo.liis.soodla@ut.ee

**Supplementary Interactive Figure 1**

*Overlap between personality-only LPA Model 1 and the k-means model, LPA Model 2, LPA Model 3 and LPA Model 4*

*Note for interactive graph.* Upper left: overlap between Model 1 profiles and clusters. Upper right: overlap between Model 1 profiles and Model 2 profiles. Lower left: overlap between Model 1 profiles and Model 3 profiles. Lower right: overlap between Model 1 profiles and Model 4 profiles.

**Supplementary Interactive Figure 2**

*Overlap between symptoms-included LPA Model 1 and the k-means model, LPA Model 2, LPA Model 3 and LPA Model 4*

**

*Note for interactive graph.* Upper left: overlap between Model 1 profiles and clusters. Upper right: overlap between Model 1 profiles and Model 2 profiles. Lower left: overlap between Model 1 profiles and Model 3 profiles. Lower right: overlap between Model 1 profiles and Model 4 profiles.
